# Confident and sensitive phosphoproteomics using combinations of collision induced dissociation and electron transfer dissociation^☆^

**DOI:** 10.1016/j.jprot.2014.03.010

**Published:** 2014-05-30

**Authors:** Mark O. Collins, James C. Wright, Matthew Jones, Julian C. Rayner, Jyoti S. Choudhary

**Affiliations:** a Proteomic Mass Spectrometry, The Wellcome Trust Sanger Institute, Hinxton, Cambridge CB10 1SA, UK; b Malaria Programme, The Wellcome Trust Sanger Institute, Hinxton, Cambridge CB10 1SA, UK

**Keywords:** CID, collision induced dissociation, ECD, electron capture dissociation, ETD, electron transfer dissociation, ETcaD, ETD with (supplemental activation), FDR, false discovery rate, FLR, false localisation rate, IMAC, immobilised metal affinity chromatography, LC, liquid chromatography, MS, mass spectrometry, MS/MS, tandem mass spectrometry, PEP, posterior error probability, PSM, peptide spectrum match, SCX, strong cation exchange, Phosphoproteomics, Phosphorylation, Mass spectrometry, Post-translational modifications, Electron transfer dissociation, *Plasmodium falciparum*

## Abstract

We present a workflow using an ETD-optimised version of Mascot Percolator and a modified version of SLoMo (turbo-SLoMo) for analysis of phosphoproteomic data. We have benchmarked this against several database searching algorithms and phosphorylation site localisation tools and show that it offers highly sensitive and confident phosphopeptide identification and site assignment with PSM-level statistics, enabling rigorous comparison of data acquisition methods. We analysed the *Plasmodium falciparum* schizont phosphoproteome using for the first time, a data-dependent neutral loss-triggered-ETD (DDNL) strategy and a conventional decision-tree method. At a posterior error probability threshold of 0.01, similar numbers of PSMs were identified using both methods with a 73% overlap in phosphopeptide identifications. The false discovery rate associated with spectral pairs where DDNL CID/ETD identified the same phosphopeptide was < 1%. 72% of phosphorylation site assignments using turbo-SLoMo without any score filtering, were identical and 99.8% of these cases are associated with a false localisation rate of < 5%. We show that DDNL acquisition is a useful approach for phosphoproteomics and results in an increased confidence in phosphopeptide identification without compromising sensitivity or duty cycle. Furthermore, the combination of Mascot Percolator and turbo-SLoMo represents a robust workflow for phosphoproteomic data analysis using CID and ETD fragmentation.

**Biological significance:**

Protein phosphorylation is a ubiquitous post-translational modification that regulates protein function. Mass spectrometry-based approaches have revolutionised its analysis on a large-scale but phosphorylation sites are often identified by single phosphopeptides and therefore require more rigorous data analysis to unsure that sites are identified with high confidence for follow-up experiments to investigate their biological significance. The coverage and confidence of phosphoproteomic experiments can be enhanced by the use of multiple complementary fragmentation methods. Here we have benchmarked a data analysis pipeline for analysis of phosphoproteomic data generated using CID and ETD fragmentation and used it to demonstrate the utility of a data-dependent neutral loss triggered ETD fragmentation strategy for high confidence phosphopeptide identification and phosphorylation site localisation.

## Highlights

• We report and benchmark a data analysis pipeline for phosphoproteomic data analysis.
• Combined use of Mascot Percolator and turbo-SLoMo to compare fragmentation methods
• CID and ETD fragmentation for phosphorylation site identification
• Demonstrate the utility of data-dependent neutral loss triggered ETD fragmentation
• High confidence of phosphoproteomic analysis using ETD/CID spectral pairs

## Graphical abstract

**Figure:**
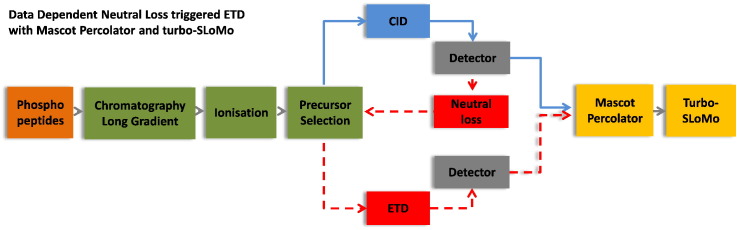

## 1 Introduction

Protein phosphorylation is the most studied post-translational modifications and it regulates most biological processes. A large proportion of genomes encode proteins that regulate protein phosphorylation (kinases and phosphatases) as well as many classes of protein domains that recognize it as a regulated epitope. The analysis of protein phosphorylation has been transformed in recent years with the development of methods to enrich phosphopeptides from complex mixtures [1–5], soft peptide fragmentation techniques such as electron transfer dissociation [6], as well as advances in the sensitivity and specificity of mass spectrometry instrumentation. Protein phosphorylation was the first PTM studied on a proteome scale and since then protein acetylation [7], ubiquitination [8] and O-GlcNAcylation [9], amongst others have benefitted from similar technological developments.

CID fragmentation is generally the method of choice for peptide fragmentation and its implementation in ion trap and Q-ToF type devices allows rapid and sensitive peptide identification. Recent generations of mass spectrometers have also been enabled with various configurations of ETD sources [6,10] enabling a type of peptide fragmentation that is considered complementary to CID [11]. CID fragmentation of peptides bearing labile modifications often result in spectra that are dominated by large neutral loss peaks [12] with compromised sequence information compared to spectra from non-modified counterparts [13]. ETD however, preserves labile side chain modifications such as phosphorylation and O-GlcNAcylation and has attracted much attention for the analysis of PTMs [14]. Generally, CID fragmentation is more suited to lower charge states and higher precursor mass, whilst ETD is better suited to higher charge but lower m/z precursor ions [15]. The duty cycle of ETD is currently slower than CID due to longer reaction times, 70–100 ms for ETD versus 10–30 ms for CID. Therefore, the combined/targeted use of these complementary methods in the same experiment can improve peptide and protein identification rates and reduce the amount of sample needed and total acquisition time [15]. Recently, it has been reported that through optimisation of ETD reaction parameters and the use of alternative reagents, ETD fragmentation can be performed at optimal efficiency in time scales similar to CID [16]. This will further enhance the use of combined alternative fragmentation methods as duty cycle constraints are lessened.

Analysis of data acquired using alternative fragmentation methods and from peptides bearing post-translational modifications is not straightforward for a number of reasons. Alternative fragmentation methods can require additional processing and an increased number of fragment ion types to be considered. This leads to a higher computational overhead with an increased risk of false positive matches. The majority of database searching algorithms were built to analyse data from CID spectra and over the years as alternative fragmentation methods have been developed, they have been modified to allow for the different ion series produced by these methods. This integration of different fragmentation types has also been implemented in post-search algorithms such as Mascot Percolator [17,18] and Protein Prospector [19], allowing scoring and accurate FDR calculations to be determined in a single pipeline regardless of fragmentation type.

For modified peptides, the search space is inflated due to additional variable modifications and an increased number of missed cleavages caused by phosphorylation sites adjacent to enzymatic cleavage sites [20]. Furthermore, phosphopeptide spectra tend to have added complexity, especially from CID, where neutral loss peaks are present. This can cause a substantial decrease in informative fragment ions and this in turn affects peptide spectrum match (PSM) scoring; modified spectra score lower than their unmodified counterparts. Again, post-search tools such as Mascot Percolator can help to boost the scores of these spectra; however this is still reliant on the initial search being able to correctly assign the peptide sequence. Finally, localisation of phosphorylation sites using fragmentation information is not straightforward [21]. Sites can only be assigned if fragment ions surrounding potential phosphorylation sites are present; often this is further complicated by multiple phosphorylatable residues being adjacent and single peptides having multiple modifications. There are various tools to help evaluate modification site assignment including Mascot Delta Score [22], Ascore [23], PhosphoRS [24] and SloMo [25] amongst others (see [21] for detailed review). These tools have been benchmarked using data from phosphopeptide standards and it is assumed that false localisation rates are consistent between standard and biological datasets [21].

We set out to explore workflows for large scale phosphoproteomics using combinations of CID and ETD fragmentation and evaluation of a single data analysis pipeline comprising of Mascot Percolator and a modified version of the site localisation tool, SLoMo (turbo-SLoMo). We investigate the performance of two types of targeted CID and ETD analysis; decision tree (DT) and data-dependent neutral loss triggered ETD (DDNL), for the analysis of the *Plasmodium falciparum* schizont phosphoproteome. We explore the utility of CID/ETD spectral pairs from DDNL experiments for high confidence phosphopeptide identification and phosphorylation site localisation, exploiting self-validating spectral pairs to assess the performance of the data analysis workflow. Finally, we use the combined Mascot Percolator and turbo-SLoMo data analysis workflow to generate a high confidence *P. falciparum* schizont phosphoproteome.

## 2 Experimental procedures

### 2.1 Preparation of parasites

*P. falciparum* strain 3D7 was cultured in 2.5–5% O + human erythrocytes with 0.5% Albumax II in custom-made RPMI media (Invitrogen) and parasites were collected by saponin lysis, an approach that removes the vast majority of erythrocyte material. Briefly, infected erythrocytes were pelleted and re-suspended in 5–10 ml of 0.1% Saponin lysis buffer (0.1% Saponin in PBS) and incubated at room temperature for 5 min before being centrifuged at 3200 ×*g* for 10 min. After centrifugation, the supernatant was discarded and the parasite pellet was washed with 0.1% Saponin lysis buffer and centrifuged again at 3200 ×*g* for 10 min. Washes with 0.1% Saponin lysis buffer were repeated until supernatants were completely clear.

### 2.2 Protein extraction, digestion and clean up

Parasite pellets were re-suspended in extraction buffer (4% SDS, 0.1 M DTT, 0.1 M Tris pH 8, 0.5 mM PMSF, 2 μg/ml Aprotinin/Leupeptin, 20 μM ZnCl and 25 mM Sodium fluoride), homogenised in a 2 ml dounce homogeniser with 25 strokes and DNA was sheared by passing the lysate through a fine gauge needle. The sample was heated for 5 min @ 97 °C and insoluble material was pelleted by centrifugation at 14,000 rpm for 10 min. The insoluble pellet was further extracted by addition of urea (once cooled to room temperature) to a final concentration of 8 M. Homogenisation and centrifugation steps were repeated and supernatants containing solubilized protein from both extractions were pooled and applied to a pre-washed Amicom-15 centrifugal filter unit (30 kD MWCO) (Millipore). The protein sample (4 mg) was processed according to the FASP procedure [26,27] in which SDS was removed by buffer exchange with urea and cysteine alkylation was performed in situ with iodoacetamide and proteins were digested with Trypsin Gold (Promega) for 4 h at 37 °C at an enzyme substrate ratio of 1:20 with a urea concentration of 1.8 M and 100 mM ammonium bicarbonate. Peptides were collected by centrifugation and addition of 100 mM ammonium bicarbonate to the upper chamber of the unit and further centrifugation. Collected peptides were adjusted to a 0.4% TFA and desalted using a Sep-Pak® Light C18 cartridge (Waters) and dried down using a SpeedVac (Thermo Scientific).

### 2.3 IMAC purification

IMAC purifications were performed as described [28], with the following modifications. Peptides were re-suspended in IMAC loading buffer (50% acetonitrile, 0.1% TFA) and incubated with pre-equilibrated Phos-Select beads (Sigma) for 1 h at room temperature. The beads were then transferred to a TopTip (Glygen) and washed once with IMAC loading buffer, 1% acetic acid and then water. Phosphopeptides were eluted with 100 μl ammonia water pH 11 and acidified using formic acid. Phosphopeptides from two IMAC purifications were pooled and split into 6 aliquots for LC–MS/MS analysis.

### 2.4 LC–MS/MS analysis

Phosphopeptide samples were analysed online using an Ultimate 3000 nano/Capillary LC System (Dionex) coupled to an LTQ Orbitrap Velos hybrid mass spectrometer (Thermo Scientific) equipped with a nanospray ion source. Peptides were desalted on-line using a micro-Precolumn cartridge (C18 Pepmap 100, LC Packings) (with 0.5% acetic acid) and then separated using a 320 min RP gradient (4–30% acetonitrile/0.1% formic acid) on an Acclaim PepMap100 C18 analytical column (3 μm, 75 μm id × 50 cm) (Dionex) with a flow rate of 0.3 μl/min. The mass spectrometer was operated in standard data dependent acquisition mode controlled by Xcalibur 2.1. The instrument was operated with a cycle of one MS (in the Orbitrap) acquired at a resolution of 60,000 at m/z 400, with the top 10 most abundant multiply-charged (2 + and higher) ions in a given chromatographic window subjected to either CID or ETD fragmentation in the linear ion trap with supplemental activation enabled. An FTMS target value of 1e6 and an ion trap MSn target value of 1e4 were used. Dynamic exclusion was enabled with a repeat duration of 45 s with an exclusion list of 500 and exclusion duration of 30 s. Lock mass of 445.120025 was enabled for all experiments. Triplicate DT experiments were performed using the standard charge and mass/charge settings in the instrument method file. Triplicate DDNL experiments were performed when neutral loss peaks corresponding to the loss of phosphoric acid from precursor ions in CID were observed within the top 5 most intense CID peaks. We also performed proteome profiling experiments (1 μg of desalted FASP digest) using direct LC–MS/MS analyses; triplicate experiments using CID fragmentation and triplicate DT experiments with 4-hour LC gradients using the same LC setup and acquisition parameters described above.

### 2.5 Data processing and database searching

All raw MS data files were processed and converted into MGF file format using Proteome Discoverer 1.1 (Thermo Scientific). A precursor filter of 600–10,000 Da and a non-fragment filter were applied to ETD spectra to remove un-reacted precursor peaks, charge reduced precursor peaks, neutral losses from charge reduced precursors and FT Overtones using default settings. All ion trap spectra with less than 15 fragmentation peaks were removed and a signal to noise filter of 3 was applied to all spectra. All datasets were searched using Mascot v2.2 (Matrix Science) against a combined Human (IPI, 2010) and *P. falciparum* (GeneDB) sequence database (79,637 sequences) using the following search parameters: trypsin with a maximum of 3 missed cleavages, 10 ppm for MS mass tolerance, 0.5 Da for MS/MS mass tolerance, with Acetyl (Protein N-term), Oxidation (M), Deamidated (NQ), Carbamidomethyl (C) and Phospho (ST) set as variable modifications. ETD spectra were searched using c, z and y ion series and CID data was searched using b and y ion series. All searches used Mascot's automated decoy database searching. All data was further analysed using Mascot Percolator V2 (http://www.sanger.ac.uk/resources/software/mascotpercolator/) [18] to re-score PSMs and generate final datasets with initially a *q*-value of 0.01 and then a posterior error probability (PEP) threshold of 0.01 was applied to the data to generate a higher confidence dataset with a less than 1% FDR at the PSM level. Only top ranked hits were reported. Phosphorylation site localisation was performed using an in-house version of SLoMo [25], Ascore [23] and the Mascot Delta score [22]. To allow high-throughput analysis of the data several in-house changes were made to the original SLoMo modification site localisation tool to generate our version of the software, turbo-SLoMo (http://www.sanger.ac.uk/resources/software/). SLoMo was adapted to read spectra in MGF format, and to also generate a single combined output results summary file. New command line options were added to allow all or a subset of modifications (such as all phosphorylation modifications) to be processed in a single run. Options were also added to run the tool in a batch mode that allowed a single analysis to be split over many computational nodes. Finally, we also added additional filtering to exclude spectra with low Mascot Percolator PEP scores and with large numbers of variable modifications. Mascot delta scores were calculated as the ion score difference between rank 1 PSMs and the next same sequence rank ion score within the top 10 ranks reported by Mascot. In cases where an additional same sequence rank hit was not found, a delta score could not be calculated and therefore was not considered a PSM with a localised phosphorylation site. Delta score thresholds for CID and ETD (with supplemental activation) equating to 1% and 5% false localisation rates were taken from [22]. CID and ETD spectra from DT experiments and CID spectra from DDNL experiments were also searched with the addition of Phospho Y to identify putative tyrosine phosphorylation sites. PSMs (PEP < 0.01) with confidently localised tyrosine phosphorylation sites (5% FLR) were accepted and in cases where a spectrum also matched an alternative Phospho (ST) site then the PSM with the highest turbo-SLoMo score was chosen and the score difference between potential pY and pST sites included in Table S6. Mascot dat files were converted to PRIDE XML files with PRIDE Converter v2.0.9 and uploaded to the ProteomeXchange Consortium (http://proteomecentral.proteomexchange.org) via the PRIDE partner repository [29] with the dataset identifier PXD000070 and DOI http://dx.doi.org/10.6019/PXD000070 and available in the PRIDE database (http://www.ebi.ac.uk/pride/) with accession numbers 27915-27926.

All raw MS data files were processed and converted into MGF file format using Proteome Discoverer 1.1 (Thermo Scientific). A precursor filter of 600–10,000 Da and a non-fragment filter were applied to ETD spectra to remove un-reacted precursor peaks, charge reduced precursor peaks, neutral losses from charge reduced precursors and FT Overtones using default settings. All ion trap spectra with less than 15 fragmentation peaks were removed and a signal to noise filter of 3 was applied to all spectra. All datasets were searched using Mascot v2.2 (Matrix Science) against a combined Human (IPI, 2010) and *P. falciparum* (GeneDB) sequence database (79,637 sequences) using the following search parameters: trypsin with a maximum of 3 missed cleavages, 10 ppm for MS mass tolerance, 0.5 Da for MS/MS mass tolerance, with Acetyl (Protein N-term), Oxidation (M), Deamidated (NQ), Carbamidomethyl (C) and Phospho (ST) set as variable modifications. ETD spectra were searched using c, z and y ion series and CID data was searched using b and y ion series. All searches used Mascot's automated decoy database searching. All data was further analysed using Mascot Percolator V2 (http://www.sanger.ac.uk/resources/software/mascotpercolator/) [18] to re-score PSMs and generate final datasets with initially a *q*-value of 0.01 and then a posterior error probability (PEP) threshold of 0.01 was applied to the data to generate a higher confidence dataset with a less than 1% FDR at the PSM level. Only top ranked hits were reported. Phosphorylation site localisation was performed using an in-house version of SLoMo [25], Ascore [23] and the Mascot Delta score [22]. To allow high-throughput analysis of the data several in-house changes were made to the original SLoMo modification site localisation tool to generate our version of the software, turbo-SLoMo (http://www.sanger.ac.uk/resources/software/). SLoMo was adapted to read spectra in MGF format, and to also generate a single combined output results summary file. New command line options were added to allow all or a subset of modifications (such as all phosphorylation modifications) to be processed in a single run. Options were also added to run the tool in a batch mode that allowed a single analysis to be split over many computational nodes. Finally, we also added additional filtering to exclude spectra with low Mascot Percolator PEP scores and with large numbers of variable modifications. Mascot delta scores were calculated as the ion score difference between rank 1 PSMs and the next same sequence rank ion score within the top 10 ranks reported by Mascot. In cases where an additional same sequence rank hit was not found, a delta score could not be calculated and therefore was not considered a PSM with a localised phosphorylation site. Delta score thresholds for CID and ETD (with supplemental activation) equating to 1% and 5% false localisation rates were taken from [22]. CID and ETD spectra from DT experiments and CID spectra from DDNL experiments were also searched with the addition of Phospho Y to identify putative tyrosine phosphorylation sites. PSMs (PEP < 0.01) with confidently localised tyrosine phosphorylation sites (5% FLR) were accepted and in cases where a spectrum also matched an alternative Phospho (ST) site then the PSM with the highest turbo-SLoMo score was chosen and the score difference between potential pY and pST sites included in Table S6. Mascot dat files were converted to PRIDE XML files with PRIDE Converter v2.0.9 and uploaded to the ProteomeXchange Consortium (http://proteomecentral.proteomexchange.org) via the PRIDE partner repository [29] with the dataset identifier PXD000070 and DOI http://dx.doi.org/10.6019/PXD000070 and available in the PRIDE database (http://www.ebi.ac.uk/pride/) with accession numbers 27915-27926.

## 3 Results and discussion

### 3.1 A data analysis workflow for phosphopeptide characterisation using CID and ETD fragmentation

**Fig. 1:**
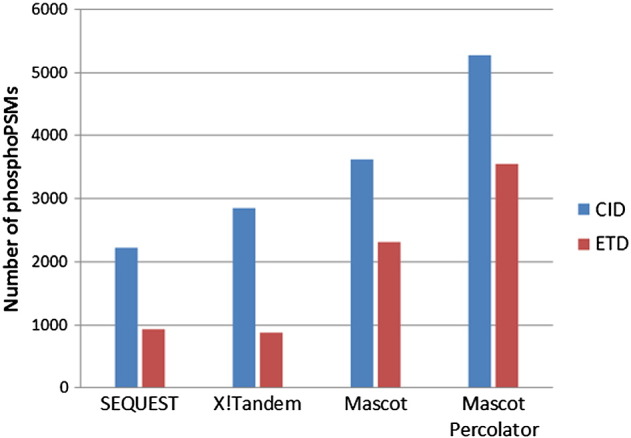
Comparison of search algorithms for identification of phosphopeptides using CID and ETD fragmentation. Numbers of phosphoPSMs identified using Sequest, X!Tandem, Mascot and Mascot Percolator for a dataset of sequential CID and ETD spectra are shown at a 1% FDR.

In order to obtain robust peptide identifications using multiple fragmentation techniques, data should be processed, database searched and scored using a single robust, global and PSM specific FDR analysis pipeline. We have recently adapted Mascot Percolator, a machine learning method for rescoring database search results from ETD [18] as well as CID data [17]. Mascot Percolator increases the number of CID PSMs by up to 80% and ETD PSMs by up to 60% at a 0.01 *q*-value (1% FDR) threshold over a standard Mascot search [18]. We benchmarked Mascot Percolator's performance to other database search and scoring algorithms for phosphopeptide identification. Details of the extended feature set that we use for SVM training in our implementation of Mascot Percolator can be found here [18]. We reanalysed published human phosphoproteomic data (phosphopeptides enriched from a human cell line), which was acquired by sequential CID and ETD analysis on an Orbitrap mass spectrometer [30], using Mascot Percolator. 5277 and 3543 phosphopeptide PSMs (0.01 *q*-value threshold) were identified for CID and ETD datasets, respectively (Fig. 1). This represents an increase in significant assignments of 45.5% and 53.1% compared to Mascot, 137.2% and 280.9% compared to SEQUEST and 84.8% and 304.5% compared to X!Tandem for CID and ETD data, respectively. The gain in phosphopeptide identifications using Mascot Percolator for both CID and ETD data is substantial however, the performance of Mascot Percolator for phosphopeptide data is slightly lower than we previously reported for data from unmodified peptides (22) reflecting the increased complexity of spectra from modified peptides. Whilst we would expect improvement in the overall performance of SEQUEST and X!Tandem when hyphenated with percolator [31,32], it is important to note that Mascot (and Mascot Percolator) performs significantly better than SEQUEST and X!Tandem for analysis of ETD data, a feature that is particularly important if CID/ETD spectral pairs are to be analysed.

In order to assess the accuracy of Mascot Percolator for CID and ETD phosphopeptide data, we processed data from a human phosphopeptide library that was analysed using CID and ETD on an Orbitrap mass spectrometer [22]. 99.2% of ETD spectra and 99.4% of CID spectra identified by Mascot Percolator (at a 0.01 PEP threshold) were matched to the phosphopeptide library and some additional common contaminant proteins (e.g. BSA, keratin, and trypsin). This equates to a false discovery rate of well below 1% which is similar to that reported for unmodified peptides [18].

**Fig. 2:**
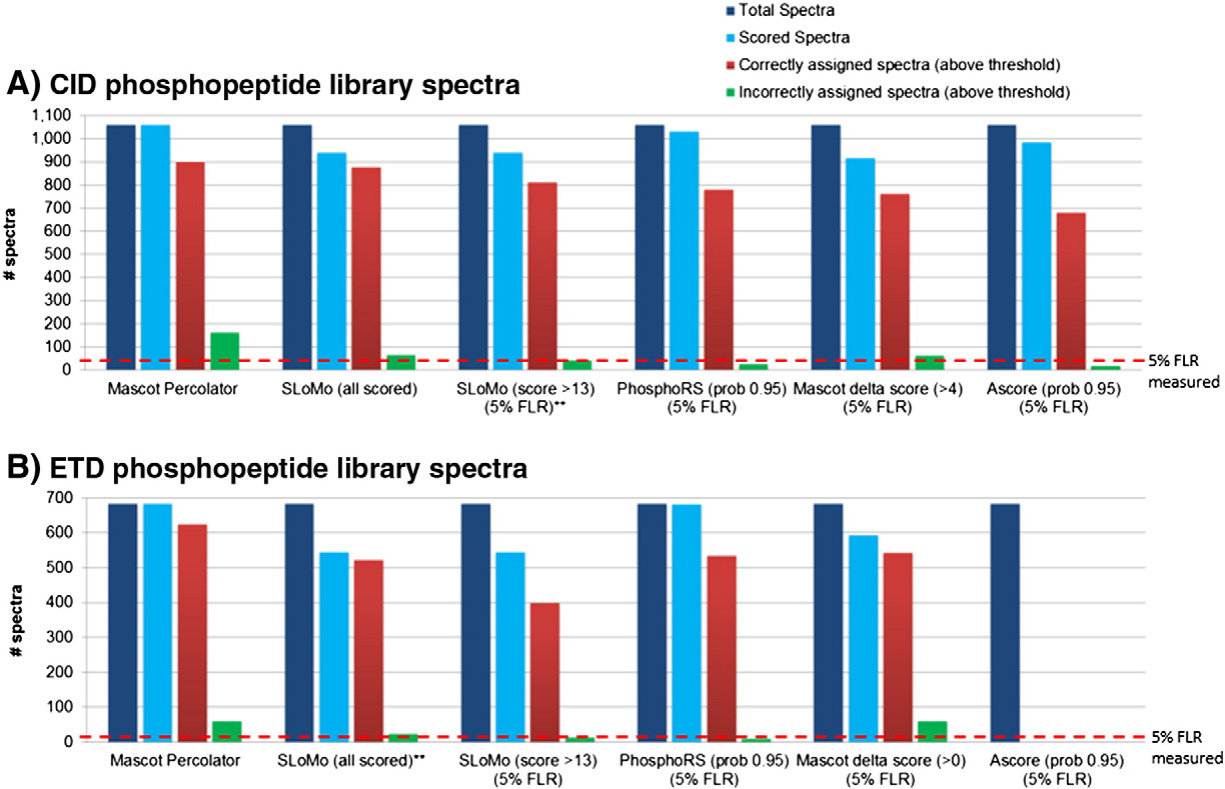
Comparison of phosphorylation site assignment methods. The performance of Mascot (default rank 1 assignment), Mascot delta score, Ascore, PhosphoRSand turbo-SLoMo (with a range of score thresholds) for phosphorylation site assignment was tested using a human phosphopeptide library analysed by both CID (A) and ETD (B) [22]. The specificity and sensitivity of each approach can be compared by looking at the proportion of incorrectly assigned spectra (green) and correctly assigned spectra (red) to the total spectra (dark blue) and the percentage of those scored (light blue). The measured false localisation rate of 5% is indicated by a dashed red line can be compared to score thresholds reported to give an FLR of 5%. Whilst Mascot (MP 1% FDR) is the most sensitive it has the highest FLR, the Mascot delta score exhibits high specificity but low sensitivity. Overall it can be seen that ETD spectra tend to give a lower false localisation rate than CID spectra and this can be exploited by using a lower score threshold for site assignment of ETD spectra (all scored, score 2.2) by turbo-SLoMo. The use of adjusted SLoMo scores in turbo-SLoMo increases performance to that of the commercial software PhosphoRS.

We next compared a number of phosphorylation site localisation tools by benchmarking them using the same phosphopeptide library dataset [22]. We began by assessing the false localisation rate (FLR) associated with phosphorylation site assignments made by Mascot (the assignment of phosphorylation sites in rank 1 peptides). For the 1062 CID spectra and 685 ETD spectra (phosphoPSMs) identified at 0.01 PEP threshold by Mascot Percolator (Fig. 2), 894 (84.73%) CID PSMs and 624 (91.36%) of ETD PSMs identified the correct site of phosphorylation indicating that phosphorylation sites are more readily localised in ETD spectra. However, in order to reach an acceptable FLR of 5%, an increase in specificity of assignment of 10% and 5% over the standard Mascot assignment is needed for CID and ETD spectra, respectively.

Spectra corresponding to high confidence phosphopeptide identifications (0.01 PEP threshold) were analysed using the Mascot Delta Score, Ascore (CID only), PhosphoRS and our modified version of SLoMo (turbo SLoMo, see Section 2.5) and the results are summarized in Figs. 2 and S1. Initially, score thresholds that have been reported to give a FLR of 1% were used (Fig. S1). At a Mascot delta score threshold (ion score difference between rank 1 and the next same sequence rank peptide) of 11, the FLR was 2.77% for CID and at a score threshold of 4; the FLR was 2.74% for ETD spectra. The Ascore probability threshold of 0.99 gave an FLR of 1.88% for CID spectra and ETD spectra could not be analysed. A PhosphoRS probability of 0.99 corresponded to a measured FLR of 0.92% for CID spectra and 1.82% for ETD spectra. Lastly, at a turbo-SLoMo score of 19, the FLR was found to be 2.11% for CID and 1.99% for ETD spectra. All four tools gave a similar FLR of < 5% which we regard as acceptable for PTM localisation. However, the striking observation was that at these thresholds only a modest portion of spectra in some cases (42.98% of CID spectra and 67.50% of ETD spectra (Mascot Delta score), 58.91% of CID spectra (Ascore), 60.79% of CID spectra and 71.16% of ETD spectra (PhosphoRS) and 61.17% of CID spectra and 43.19% of ETD spectra (turbo-SLoMo)) were confidently localised at these thresholds which is a considerable sacrifice in sensitivity to gain modest increases in specificity over standard Mascot assignments. The observation that ETD spectra are inherently more likely to identify correct sites of phosphorylation [33] has been exploited to allow the use of a much lower Mascot delta score threshold for ETD spectra (delta score of 4 for 1% and 0 for 5% FLR) compared to CID data (delta score of 11 for 1% and 4 for 5%) with comparable false localisation rates [22] and explains the increased performance of the Mascot delta score compared to turbo-SLoMo for ETD data. We therefore investigated whether we could adjust turbo-SLoMo score thresholds to further increase the proportion of spectra for which we could confidently assign phosphorylation sites.

**Fig. 3:**
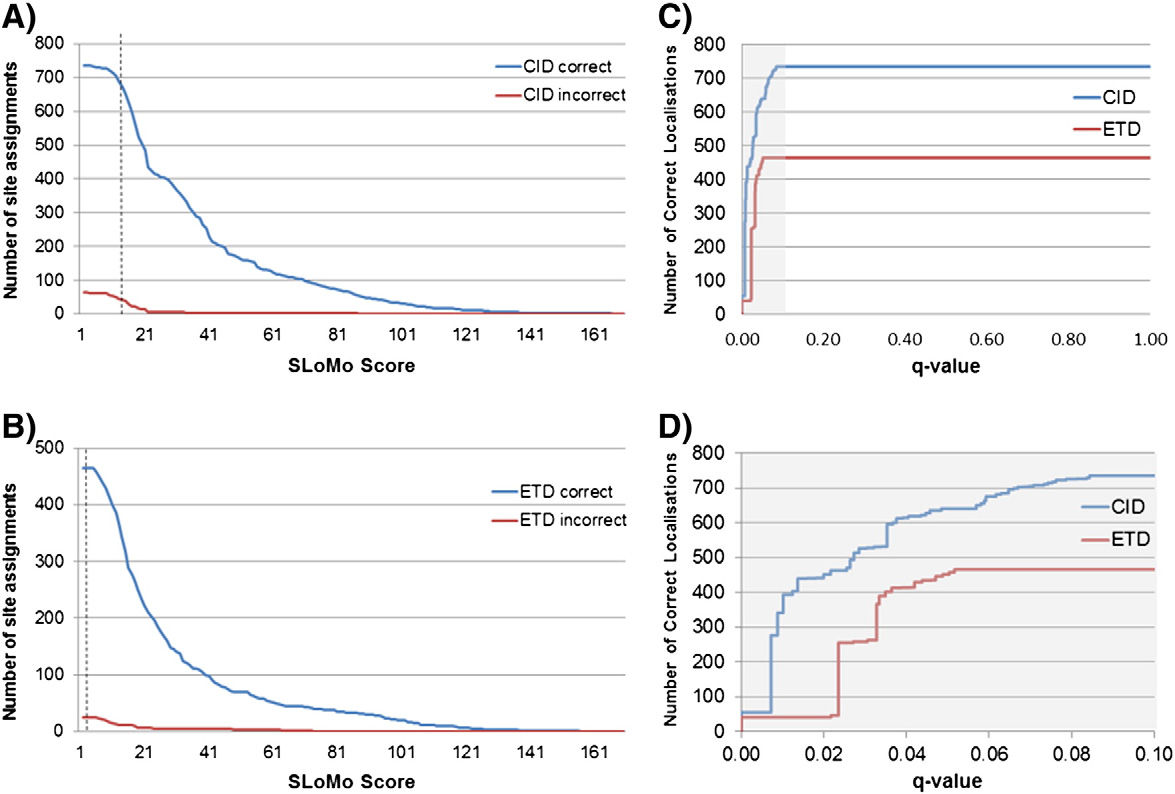
Calculation of adjusted SLoMo thresholds for CID and ETD data. The number of correct (blue line) and incorrect (red line) phosphorylation site assignments is plotted versus turbo-SLoMo score for CID data (A), ETD data (B) from the phosphopeptide library. The turbo-SLoMo score at which a 5% false localisation rate is achieved is indicated by the dashed line and is significantly higher for CID than ETD data. *q*-Value PSM plots (C and D (showing low *q*-value range)) show that the false localisation rate for turbo-SLoMo scored ETD data does not go beyond 0.05.

We found that as we dropped the score threshold from 19 that the FLR was maintained at < 5% for CID spectra until we reached a score threshold of 13 at which point 76.53% of the spectra were confidently localised (Figs. 2 and 3). Repeating the same procedure with the ETD data, we found that a much lower threshold (score of 2.2 (all scored by turbo-SLoMo)) could be used whilst still maintaining a FLR of 5% as which point 76.28% of spectra were confidently localised. The relationship between the site localisation *q*-value (FLR) and localisation rate is plotted in Fig. 3. It can be seen that whilst the FLR continues to increase with decreasing score for CID data, the FLR does not go beyond 5% for ETD data. The lowest score that turbo-SLoMo reported was 2.2, beyond this it reported a score of 0 indicating multiple top hits. Therefore, it appears that if turbo-SLoMo assigns a score to an ETD spectrum then the associated FLR will not exceed 5%. As this library contains tryptic peptides with real phosphorylation sites (they exist in nature) it is likely to be more similar to real phosphoproteomic datasets than the degenerate phosphopeptide libraries that previously had been used to optimize and test PTM localisation tools and associated false localisation rates [25]. The use of adjusted score thresholds in turbo-SLoMo to maximise site localisation whilst maintaining FLR rates of 5% significantly enhances its performance to the point where it out performs the Mascot delta score and Ascore and is comparable in performance to the leading commercially available tool, PhosphoRS.

### 3.2 Large scale phosphopeptide identification using Mascot Percolator

Next, we sought to investigate the performance of combinations of CID and ETD fragmentation for analysis of complex phosphoproteome samples. Two acquisition strategies were employed to identify phosphopeptides from IMAC enriched *P. falciparum* phosphopeptide samples. A data dependent neutral loss triggered ETD (DDNL) acquisition method in which every precursor is fragmented by CID and if a characteristic neutral loss of phosphoric acid is observed, the same precursor is also triggered for ETD analysis. In this way, CID scans act as ‘phosphopeptide filter’ to invest more time consuming ETD analysis on *bona fide* phosphopeptides. A similar approach implemented with neutral loss triggered ECD fragmentation was developed for phosphopeptide characterisation [34] but was somewhat limited by the speed and sensitivity of ECD and is therefore not suitable for phosphoproteome analysis. We compared this DDNL strategy to the decision tree method developed by Coon and co-workers [15] in which CID or ETD is used to fragment peptides depending on precursor charge and m/z (Fig. S2). Triplicate experiments were performed using both the DT and DDNL strategies in which the most abundant top 10 precursor ions were subjected to CID/ETD fragmentation. Similar numbers of fragmentation spectra were acquired for both methods with 163,820 for the DT and 159,294 for the DDNL triplicate datasets (Tables 1, S1).

Next, we sought to investigate the performance of combinations of CID and ETD fragmentation for analysis of complex phosphoproteome samples. Two acquisition strategies were employed to identify phosphopeptides from IMAC enriched *P. falciparum* phosphopeptide samples. A data dependent neutral loss triggered ETD (DDNL) acquisition method in which every precursor is fragmented by CID and if a characteristic neutral loss of phosphoric acid is observed, the same precursor is also triggered for ETD analysis. In this way, CID scans act as ‘phosphopeptide filter’ to invest more time consuming ETD analysis on *bona fide* phosphopeptides. A similar approach implemented with neutral loss triggered ECD fragmentation was developed for phosphopeptide characterisation [34] but was somewhat limited by the speed and sensitivity of ECD and is therefore not suitable for phosphoproteome analysis. We compared this DDNL strategy to the decision tree method developed by Coon and co-workers [15] in which CID or ETD is used to fragment peptides depending on precursor charge and m/z (Fig. S2). Triplicate experiments were performed using both the DT and DDNL strategies in which the most abundant top 10 precursor ions were subjected to CID/ETD fragmentation. Similar numbers of fragmentation spectra were acquired for both methods with 163,820 for the DT and 159,294 for the DDNL triplicate datasets (Tables 1, S1).

**Fig. 4:**
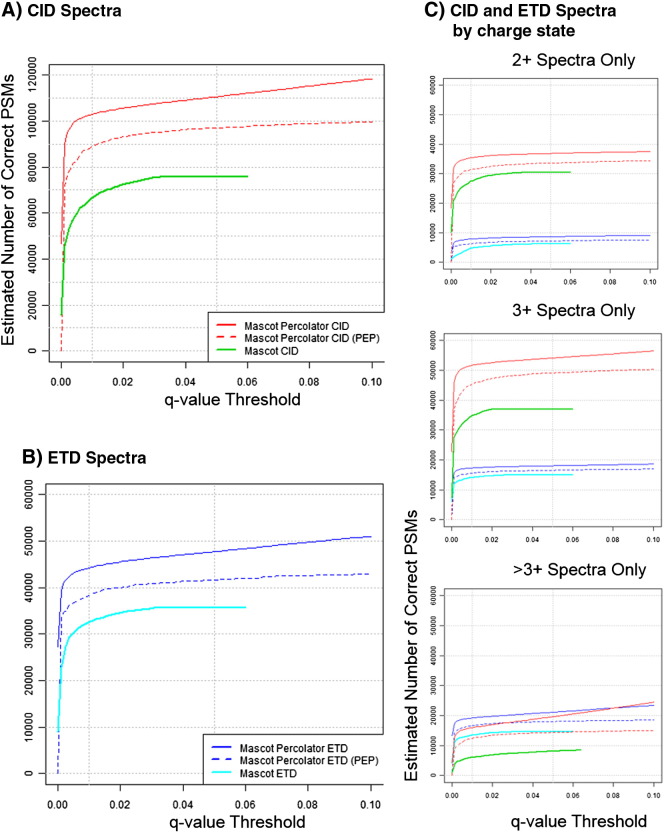
Phosphopeptide spectrum match *q*–*p* plots. These *q*-value PSM plots display the estimated number of correct PSMs for CID and ETD spectra from DT and DDNL experiments using Mascot, and Mascot Percolator across a range of *q*-value and PEP thresholds. Plots A and B show CID and ETD datasets, respectively and plots in panel C show the estimated correct PSMs for 2 +, 3 +, and > 3 + precursor charge states. Mascot Percolator gave 54.5% and 35.9% gains in PSMs at a 0.01 *q*-value (1% FDR) threshold over the standard Mascot search for CID and ETD data, respectively.

We evaluated the performance of Mascot Percolator over standard Mascot results for over 227,000 CID spectra and over 95,000 ETD spectra from the combined set of DT and DDNL experiments. At a 1% FDR (0.01 *q*-value threshold) Mascot Percolator gave an overall 54.5% increase in CID PSMs over the standard Mascot search (0.01 *q*-value threshold) with 22.6, 64.4 and 150.6% gains for 2 +, 3 + and > 3 + charges states respectively (Fig. 4, Table S1). A similar gain in matched PSMs was observed with ETD data with an overall increase of 35.9% in significant PSMs over the standard Mascot search (0.01 *q*-value threshold) with 45.6, 23.7 and 45.0% gains for 2 +, 3 + and > 3 + charge states, respectively (Fig. 4 (Table S1)). We chose to use a more conservative cut off than the usual 1% FDR (0.01 *q*-value threshold) used for protein identification as we believe that when dealing with modified peptides, PSM specific scoring should be used to ensure high confidence at the individual PSM level [35]. We therefore chose to use a 0.01 PEP threshold for reporting phosphopeptide identifications, this equates to a more conservative statistical cut-off equivalent to an overall FDR of 0.14% [36] (Table S1). It can be seen in Fig. 4 that this threshold results in approximately a 15% reduction in PSMs compared to a global FDR threshold (*q* value) of 1%.

We evaluated the performance of Mascot Percolator over standard Mascot results for over 227,000 CID spectra and over 95,000 ETD spectra from the combined set of DT and DDNL experiments. At a 1% FDR (0.01 *q*-value threshold) Mascot Percolator gave an overall 54.5% increase in CID PSMs over the standard Mascot search (0.01 *q*-value threshold) with 22.6, 64.4 and 150.6% gains for 2 +, 3 + and > 3 + charges states respectively (Fig. 4, Table S1). A similar gain in matched PSMs was observed with ETD data with an overall increase of 35.9% in significant PSMs over the standard Mascot search (0.01 *q*-value threshold) with 45.6, 23.7 and 45.0% gains for 2 +, 3 + and > 3 + charge states, respectively (Fig. 4 (Table S1)). We chose to use a more conservative cut off than the usual 1% FDR (0.01 *q*-value threshold) used for protein identification as we believe that when dealing with modified peptides, PSM specific scoring should be used to ensure high confidence at the individual PSM level [35]. We therefore chose to use a 0.01 PEP threshold for reporting phosphopeptide identifications, this equates to a more conservative statistical cut-off equivalent to an overall FDR of 0.14% [36] (Table S1). It can be seen in Fig. 4 that this threshold results in approximately a 15% reduction in PSMs compared to a global FDR threshold (*q* value) of 1%.

### 3.3 Combinations of CID and ETD for phosphopeptide identification

In the DT strategy, 163,820 spectra were acquired in three experiments (Table 1), with 45,862 CID PSMs and 18,123 ETD PSMs identified by Mascot Percolator at a 0.01 PEP threshold (Tables S1 & S3). In total, the DT method identified 3798 unique phosphopeptide sequences (0.01 PEP threshold) with 3272 identified from CID spectra and 1361 identified from ETD spectra (Table 2). The overlap in phosphopeptide identifications between CID and ETD spectra using this method was 21.99% (Table 2, Fig. S3) which is similar to that reported previously (17.9%) for phosphopeptide analysis with this method [15].

In the DT strategy, 163,820 spectra were acquired in three experiments (Table 1), with 45,862 CID PSMs and 18,123 ETD PSMs identified by Mascot Percolator at a 0.01 PEP threshold (Tables S1 & S3). In total, the DT method identified 3798 unique phosphopeptide sequences (0.01 PEP threshold) with 3272 identified from CID spectra and 1361 identified from ETD spectra (Table 2). The overlap in phosphopeptide identifications between CID and ETD spectra using this method was 21.99% (Table 2, Fig. S3) which is similar to that reported previously (17.9%) for phosphopeptide analysis with this method [15].

159,294 spectra were acquired in three DNNL experiments (Table 1), with 43,183 CID PSMs (assignment rate of 39.1%) and 20,140 (assignment rate of 41.1%) ETD PSMs identified by Mascot Percolator at a 0.01 PEP threshold (Tables S1 & S2). In total, the DDNL method identified 3808 unique phosphopeptide sequences (0.01 PEP threshold) with 3272 identified from CID spectra and 2996 identified from ETD spectra (Table 2). The overlap in phosphopeptide identifications between CID and ETD spectra for this method was 64.5% (Table 2, Fig. S3). 71% of PSMs (0.01 PEP threshold) (CID and ETD spectra) in the DT method were matched to phosphopeptides. This contrasts with an increase in phosphoPSMs to 89% for ETD spectra in the DDNL strategy which employs a phosphorylation selection step in the form of the neutral loss trigger. Overall, the DT and DDNL methods identified similar numbers of unique phosphopeptides however; given that a large proportion of the DDNL data is composed of consecutive CID and ETD spectra of the exact same precursor, confidence in phosphopeptide identification by both fragmentation methods is increased.

159,294 spectra were acquired in three DNNL experiments (Table 1), with 43,183 CID PSMs (assignment rate of 39.1%) and 20,140 (assignment rate of 41.1%) ETD PSMs identified by Mascot Percolator at a 0.01 PEP threshold (Tables S1 & S2). In total, the DDNL method identified 3808 unique phosphopeptide sequences (0.01 PEP threshold) with 3272 identified from CID spectra and 2996 identified from ETD spectra (Table 2). The overlap in phosphopeptide identifications between CID and ETD spectra for this method was 64.5% (Table 2, Fig. S3). 71% of PSMs (0.01 PEP threshold) (CID and ETD spectra) in the DT method were matched to phosphopeptides. This contrasts with an increase in phosphoPSMs to 89% for ETD spectra in the DDNL strategy which employs a phosphorylation selection step in the form of the neutral loss trigger. Overall, the DT and DDNL methods identified similar numbers of unique phosphopeptides however; given that a large proportion of the DDNL data is composed of consecutive CID and ETD spectra of the exact same precursor, confidence in phosphopeptide identification by both fragmentation methods is increased.

**Fig. 5:**
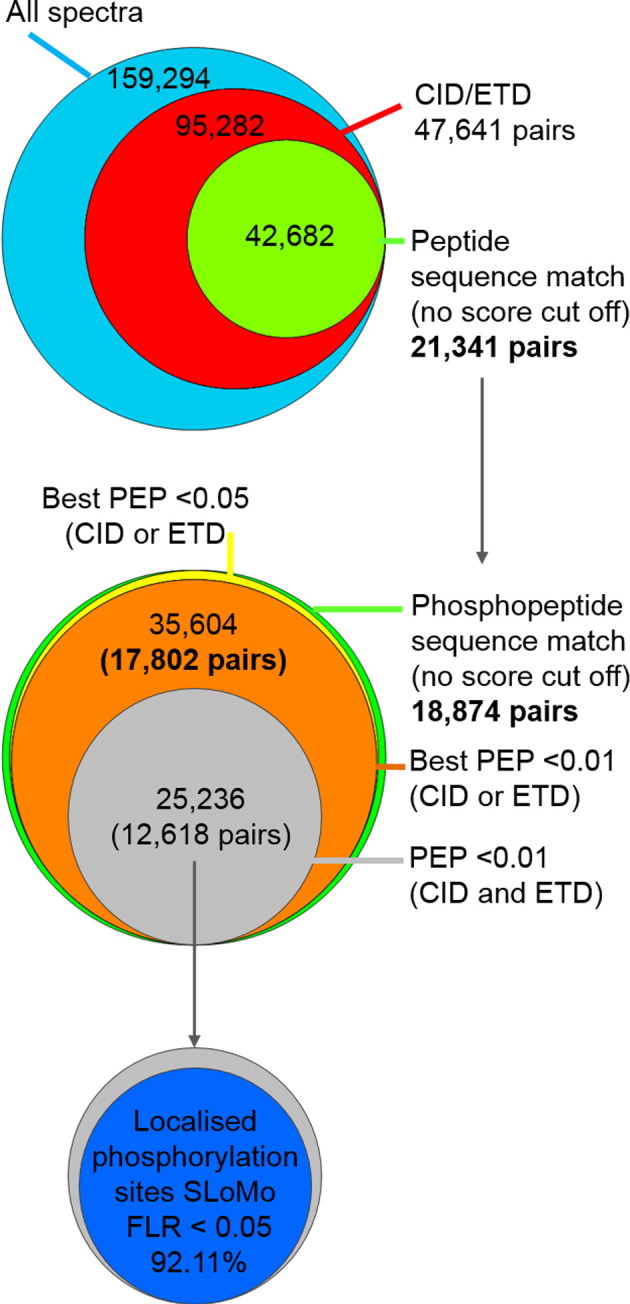
Confidence of CID and ETD spectral pair identifications in DDNL experiments. Venn diagrams showing the breakdown of spectral pairs in DDNL experiments; of the 47,641 spectral pairs acquired, 21,341 (44.8%) identified the same peptide sequence by both CID and ETD without any filtering of Mascot Percolator scores (all PSMs irrespective of peptide confidence). 88.4% of these sequence matching pairs identified phosphopeptides, 97.5% (18,407) of which had a PEP of 0.05 or less and 94.3% (17,802) had a PEP of 0.01 or less. This result indicates that if the same peptide sequence is identified by both CID and ETD in a spectral pair that the identification is highly likely to be correct as assessed by Mascot Percolator. Of 12,618 high confidence spectral pairs (PEP of 0.01 in CID and ETD, 11,622 (92.11%)) could be assigned exact sites of phosphorylation using turbo-SLoMo (FLR 0.05).

60% of the acquired spectra in DDNL experiments belonged to CID/ETD spectral pairs (95,282 spectra, 47,641 pairs) (Table 2 & Fig. 5). Of these spectral pairs, 44.8% (21,341) identified the same peptide sequence using Mascot Percolator without any score filtering. 18,874 of these spectral pairs identified the same phosphopeptide sequence and notably the majority correspond to high confidence assignments; 97.5% (18,407) had a PEP less than 0.05 and 94.3% (17,802) had a PEP less than 0.01 (Fig. 5) in at least one fragmentation method. Therefore, we observe that if the same phosphopeptide sequence is identified (no score filtering) by both CID and ETD in a spectral pair acquired in DDNL experiments, it is almost certainly a correct identification, equating to an overall dataset FDR of less than 1% without any score based filtering (0.01 *q*-value). This is also a good measure of the performance of Mascot Percolator as it confidently identified nearly all of the same phosphopeptide sequence matching spectral pairs which are highly likely to be real self-validating phosphopeptide identifications.

**Fig. 6:**
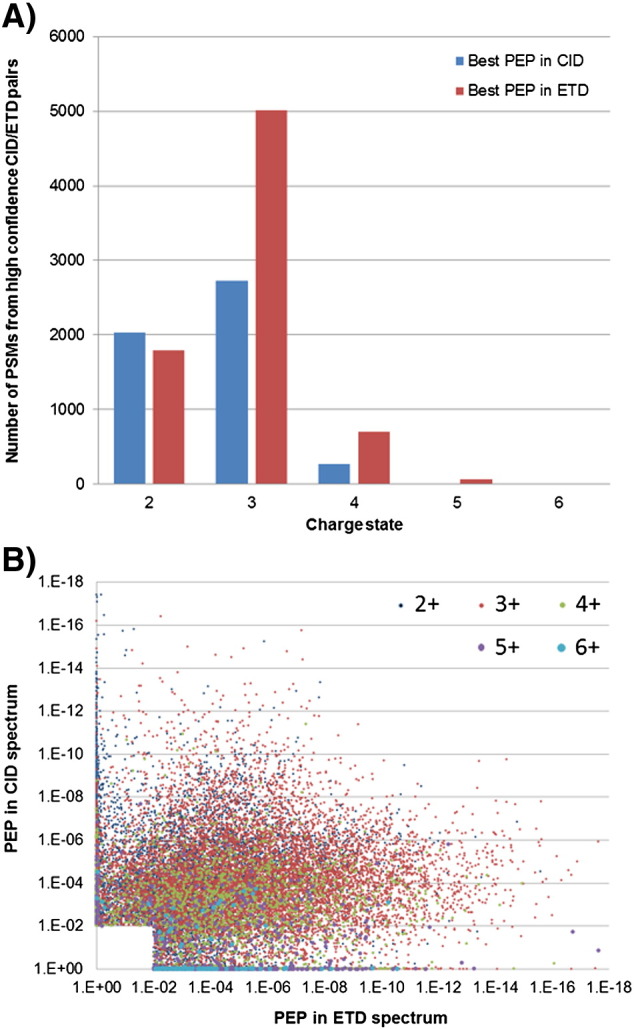
Influence of charge state on phosphopeptide identifications from CID and ETD spectral pairs. A: Numbers of CID and ETD PSMs from high confidence CID/ETD spectral pairs (both with PEP < 0.01) from DDNL experiments are plotted to show which fragmentation method provided the best PEP values for each charge state. ETD clearly results in higher confidence phosphopeptide identification for 3 + and higher charge states. B: The distribution of PEP values for spectral pairs (PEP < 0.01 in ETD or CID) is plotted and is colour coded by charge state. The overall distribution is skewed towards ETD giving significantly better PEP values than paired CID spectra.

In agreement with previous reports [15], we observed that more PSMs were identified for lower m/z and higher charge state in ETD than for CID (Fig. S4). However, the distribution of charge states for these phosphopeptides is higher than that typically observed for non-modified peptides generated using trypsin, with triply charged precursors being the most abundant. Fig. 6A compares the numbers of PSMs assigned from DDNL CID/ETD pairs using the best Mascot Percolator score of each pair. Fragmentation of doubly charged phosphopeptides gives similar numbers of best scoring PSMs by both fragmentation methods with CID performing slightly better (13% more). However, the strong bias of ETD towards triply charged phosphopeptides is striking with 84% more ETD spectra from spectral pairs scoring better then CID spectra. ETD outperformed CID for > 3^+^ charge states mainly between m/z of 500 and 800 whilst CID provides more confident identifications of doubly charged precursors across most of the m/z range and triply charged species higher than 900 m/z. The overall distribution of PEP values for spectral pairs (with a PEP of 0.01 in either CID or ETD) is plotted in Fig. 6B and is strongly skewed towards PSMs having better PEP scores from ETD spectra. The median PEP for ETD PSMs was almost two-fold smaller than that for paired CID spectra and a significant portion of spectral pairs (29%) had a PEP of 0.01 by only one fragmentation method (Fig. 6B, Table 3A). Therefore, even with the superior performance of Mascot Percolator for identification of doubly charged peptides from ETD spectra compared to OMSSA [18] (Figs. 6, S4), there is still significant complementarity between CID and ETD for phosphopeptide identification and a clear utility for combinations of these fragmentation methods for phosphoproteome analysis.

### 3.4 Phosphorylation site assignment using turbo-SLoMo

We analysed our *P. falciparum* CID and ETD datasets using turbo-SLoMo with adjusted score thresholds to give a FLR of 5%. Of the very high confidence set (0.01 PEP threshold for both) of 12,618 CID/ETD pairs identified in DDNL experiments, 10,902 (86.4%) phosphorylation site assignments (rank 1 assignment from Mascot Percolator) were identical for both fragmentation methods (Table 3a). Overall, sites in 11,622 (92.1%) high confidence spectral pairs were localised using turbo-SLoMo (FLR 5%) by either CID or ETD (Table 3b, Fig. 5). 9063 (71.8%) of these spectral pairs had the same phosphorylation site assignments using turbo-SLoMo (no score cut off applied). Strikingly, 9049/9063 (99.8%) of those spectral pairs scored by turbo-SLoMo had scores above the adjusted thresholds (5% FLR) for at least one member of CID/ETD spectral pairs. Therefore, if phosphorylation site assignments from CID/ETD pairs are the same, then they are highly likely to be correct. This mirrors the near certainty of matching peptide identifications from CID/ETD pairs that we and others [37] have observed and highlights the utility of sequential fragmentation of the same precursor by different methods. Furthermore, as almost all (99.85%) spectral pairs that were scored by turbo-SLoMo and identified the same site, were in fact localised with a FLR of 5% by either CID or ETD, there is little utility in combining site localisation scores for spectral pairs to boost site identification rates from turbo-SLoMo. Integration of site localisation scores from spectral pairs has recently been reported [38] and was shown to outperform A-score results from CID or ETD data individually but not compared to aggregate results from both CID and ETD. However, the use of an adjusted threshold for this combined score could potentially improve site localisation rates [38].

The remaining 2573 high confidence CID/ETD pairs did not give the same phosphorylation site assignment at a 5% FLR by turbo-SLoMo. The majority of the site assignments (1900, 73.8%) scored above threshold only in ETD members of the spectral pairs, again reflecting the increased utility of ETD for phosphorylation site assignments [33]. Crucially, only 258 CID/ETD pairs gave confident site assignments that were on alternative residues in the same peptide, corresponding to 2% of pairs that were analysed using turbo-SLoMo with adjusted score thresholds. In order to identify the most likely sites of phosphorylation from these CID/ETD pairs with confident but conflicting site assignments, the sites with the highest turbo-SLoMo scores were chosen. We sought to verify if this approach was valid by checking if any of these sites could be confirmed by other confidently assigned/localised spectra (turbo-SLoMo 0.05 in DT or DDNL experiments) and we found that 237/258 of these assignments (based on the highest turbo-SLoMo score) were validated by other spectra. Of the remaining 21 conflicting spectral pairs for which we could not validate the site assignment, 20 were assigned by a higher scoring CID spectrum and one by a higher scoring ETD spectrum pointing perhaps to the increased false localisation rate associated with assignments from CID spectra. The very small number of confidently assigned (by turbo-SLoMo) sites that are conflicting between CID and ETD spectral pairs supports the notion that there is little gas-phase relocation of phosphate groups in CID fragmentation [39,40].

In total, 11,622 (92.1%) high confidence CID/ETD pairs resulted in confident phosphorylation site assignment (Table 3a). By comparison, of 15,321 spectral pairs in which only the CID spectrum gave a confident peptide identification (0.01 PEP threshold), 7650 (49.9%) gave a confident phosphorylation site assignment. Of 5336 spectral pairs in which only the ETD spectrum gave a confident peptide identification (0.01 PEP threshold), 4146 (76.9%) gave a confident phosphorylation site assignment. For the DT experiments, application of adjusted turbo-SLoMo score thresholds resulted in confident site assignment of 17,873 (54.9%) CID spectra and 8334 (64.5%) ETD spectra (Table 3b). Overall, we have shown that ETD is better for phosphorylation assignment than CID (Table 3) at 5% FLR, if adjusted turbo-SLoMo score thresholds are used.

### 3.5 The *P. falciparum* proteome and phosphoproteome

The combined use of triplicate DT and DDNL analyses resulted in the identification of 4409 unique phosphopeptide sequences (mapping to 1225 unique phosphoproteins) with an overlap in identifications of 72.5% (Fig. S3c). This is a significant number of phosphopeptide identifications given that the *P. falciparum* genome contains only approximately 5300 genes [41] and an extensive phosphopeptide fractionation was not employed. In total, 2899 unique phosphorylation sites (0.01 PEP, 5% FLR thresholds) were localised at high confidence (Table S4). We also performed proteome profiling experiments from schizont digests (starting material that was used for phosphorylation analysis) using direct LC–MS/MS analyses; triplicate experiments using CID fragmentation and triplicate DT experiments with 4-hour LC gradients. 1857 proteins were identified in CID-only experiments and 1770 proteins identified in DT experiments with an overlap of 1630 (82%) proteins (Fig. S5 & Table S5).

The combined use of triplicate DT and DDNL analyses resulted in the identification of 4409 unique phosphopeptide sequences (mapping to 1225 unique phosphoproteins) with an overlap in identifications of 72.5% (Fig. S3c). This is a significant number of phosphopeptide identifications given that the *P. falciparum* genome contains only approximately 5300 genes [41] and extensive phosphopeptide fractionation was not employed. In total, 2899 unique phosphorylation sites (0.01 PEP, 5% FLR thresholds) were localised at high confidence (Table S4). We also performed proteome profiling experiments from schizont digests (starting material that was used for phosphorylation analysis) using direct LC–MS/MS analyses; triplicate experiments using CID fragmentation and triplicate DT experiments with 4-hour LC gradients. 1857 proteins were identified in CID-only experiments and 1770 proteins identified in DT experiments with an overlap of 1630 (82%) proteins (Fig. S5 & Table S5).

**Fig. 7:**
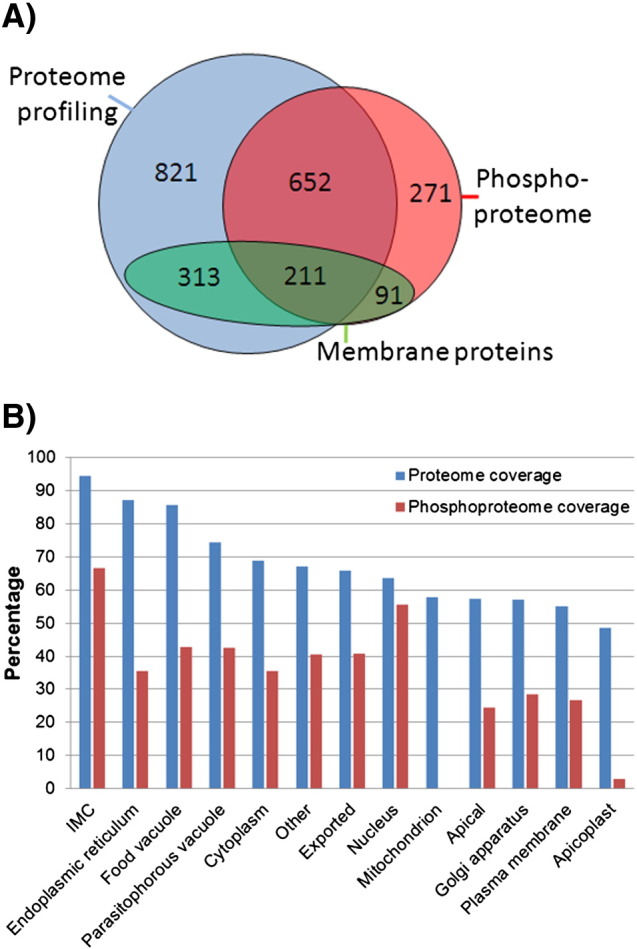
The *Plasmodium falciparum* proteome and phosphoproteome. A: Venn diagram of the overlap of proteins identified in proteome profiling experiments with phosphoproteins identified in phosphoproteomic experiments. B: The coverage of annotated subcellular locations obtained in proteome profiling and phosphoproteomic experiments is plotted. Notable differences were found in the mitochondrion and the Apicoplast which appear to have lower rates of protein phosphorylation compared to other subcellular locations.

Overall, we report identification of 2359 *P. falciparum* schizont proteins (Fig. 7), almost half of the predicted proteome. 70% of proteins identified in phosphorylation experiments were also identified in proteome profiling experiments and an additional 362 phosphoproteins (15% of total protein identifications) were only identified in the phosphorylation experiments, most likely comprising proteins expressed at low levels that are only identified after an enrichment step such as IMAC. In addition, we observed good coverage of membrane protein phosphorylation, 615 proteins with transmembrane domains were identified, 49% of which we identified with phosphorylation sites, which is a good indication that efficient protein extraction and sufficient phosphoproteome depth were achieved (Fig. 7A). Furthermore, comparison of this dataset to the palmitoylated complement of the *P. falciparum* proteome [42] reveals that 184 proteins are both phosphorylated and palmitoylated indicating potential dual regulation of these proteins at membranes.

We assessed the coverage of our proteome and phosphoproteome data in terms of experimentally annotated protein localisation using the ApiLoc database (Fig. 7B). The highest coverage (identified/annotated) was observed for inner membrane complex proteins; 95% of annotated IMC proteins were identified in proteome profiling experiments and 67% were identified in phosphorylation experiments. The IMC is a complex cytoskeletal structure that plays a role in both the development of merozoites within an infected erythrocyte and also the invasion of new host erythrocytes after merozoites are released. The enrichment of phosphorylation sites in IMC proteins implies that the IMC is a dynamically regulated organelle, with phosphorylation potentially regulating the merozoite shape changes that accompany development and invasion. The largest class of phosphoproteins was localised to the nucleus (35 proteins), closely followed by cytoplasmic (32 proteins) and exported proteins (31 proteins). We found a striking lack of phosphorylation in proteins localised to the Apicoplast (17 proteins identified, 1/17 phosphorylated) and to the mitochondrion (15 proteins identified, 0/15 proteins phosphorylated). This may be explained by the ancestral origins of these subcellular structures, in which phosphorylation is not as widespread [43,44].

Until recently [44–46], it was thought that tyrosine phosphorylation did not occur on plasmodium proteins because genes coding for tyrosine kinases could not be identified in their genomes. However, phosphoproteomic identification of putative tyrosine phosphorylation has been validated for two proteins [45]. We have also analysed our data to look for tyrosine phosphorylation and we have generated a putative list of 70 tyrosine phosphorylation sites (Table S6). Of these, four are from contaminating human proteins (all four are known phosphorylation sites) and another four were also identified as being tyrosine phosphorylated in *P. falciparum* by Treeck and co-workers [44]. Our set of 70 putative tyrosine phosphorylation sites contains 18 that were identified by multiple PSMs, and are therefore more confident and would be a good starting point for further experimental validation.

Until recently [44–46], it was thought that tyrosine phosphorylation did not occur on plasmodium proteins because genes coding for tyrosine kinases could not be identified in their genomes. However, phosphoproteomic identification of putative tyrosine phosphorylation has been validated for two proteins [45]. We have also analysed our data to look for tyrosine phosphorylation and we have generated a putative list of 70 tyrosine phosphorylation sites (Table S6). Of these, four are from contaminating human proteins (all four are known phosphorylation sites) and another four were also identified as being tyrosine phosphorylated in *P. falciparum* by Treeck and co-workers [44]. Our set of 70 putative tyrosine phosphorylation sites contains 18 that were identified by multiple PSMs, and are therefore more confident and would be a good starting point for further experimental validation.

## 4 Conclusions

The combined use of multiple fragmentation methods for analysis of complex peptide mixtures has been an attractive approach to increase proteome coverage but has been limited by appropriate data analysis pipelines to efficiently analyse and score different data types [30,37,47]. The use of Mascot Percolator to analyse CID and ETD spectral pairs allowed us to perform a direct comparison of these fragmentation techniques for large-scale phosphoproteomics. The overlap in phosphopeptide identifications for high confidence CID/ETD spectral pairs was over 70% but generally ETD produced PSMs with significantly better PEP scores than paired CID spectra. Although, both fragmentation methods can lead to confident identification of the same phosphopeptide in many cases, confident assignment of phosphorylation sites benefits from acquisition of spectral pairs and in particular fragmentation by ETD. The use of merged CID/ETD spectra [30] and hybrid fragmentation techniques such as EThcD [47,48] offers potential routes to maximise complementarity of alternative fragmentation methods, particularly for phosphorylation site localisation [48]. These approaches are currently limited by increased spectral complexity and the increased number of ion series necessary for database searching which ultimately require increased score thresholds necessary to maintain false discovery rates.

Targeted analysis of phosphopeptides by CID and ETD as implemented in the DDNL strategy allows acquisition of spectral pairs that self-validate both at the level of phosphopeptide identification and phosphorylation site localisation. This extra confidence comes without loss in sensitivity and represents a straightforward approach for stringent phosphoproteome analysis. Importantly, data from DDNL experiments allowed us to validate our data analysis workflow (using data from biological samples) though using self-validation of identifications and phosphorylation site assignments from spectral pairs. We conclude that Mascot Percolator and turbo-SLoMo are ideally suited for large scale analysis of protein phosphorylation using CID and ETD fragmentation.

The following are the supplementary data related to this article.

Supplementary Figures.

Table S1 Summary of all data acquired in DT and DDNL experiments. Numbers of PSMs (A) and unique peptides (B) identified by Mascot Percolator (0.01, 0.05 PEP and 0.01, 0.05 q-value thresholds) and Mascot (Mascot homology threshold (MHT) 0.01, 0.05 q-value threshold) are summarised for individual replicates of CID and ETD data from DDNL and DT experiments and are divided into charge state bins. Note that these numbers are total numbers and non-phosphorylated peptides are not filtered away.

Table S2 All DDNL phosphoPSMs. A: Identified by CID/ETD spectral pairs at a 0.01 PEP threshold in both fragmentation types. B: Identified by ETD only at 0.01 PEP threshold. C: Identified by CID only at a 0.01 PEP threshold. In all three sheets, the results of phosphorylation site assignment by SLoMo for each PSM are included and in sheet A additional columns indicating whether identified sequences and phosphorylation sites for CID/ETD pairs matched between fragmentation types are included. Putative tyrosine phosphorylation sites are indicated (CID spectra only) and the difference in SLoMo score between a phosphotyrosine site localisation and an alternative phosphoserine/ threonine localisation is included. ETD spectra from DDNL experiments were not searched for the presence of tyrosine phosphorylation as it is not expected that neutral losses from tyrosine phosphorylated peptides would occur and therefore ETD fragmentation would not be triggered. All PSMs matching to contaminating human proteins are coloured in red font. CID spectra can be identified by a white background whilst ETD spectra have a grey background.

Table S3 All DT phosphoPSMs. A: Identified by CID at a 0.01 PEP threshold. B: Identified by ETD at 0.01 PEP threshold. In both sheets, the result of phosphorylation site assignment by SLoMo for each PSM is included. Putative tyrosine phosphorylation sites are indicated (for CID and ETD spectra) and the difference in SLoMo score between a phosphotyrosine site localisation and an alternative phosphoserine/threonine localisation is included. All PSMs matching to contaminating human proteins are coloured in red font. CID spectra can be identified by a white background whilst ETD spectra have a grey background.

Table S4 All localised phosphorylation sites. All phosphorylation sites on unique phosphopeptides identified with a 0.01 PEP threshold and localised with a false localisation rate of 5% are included.

Table S5 *Plasmodium falciparum* proteome profiling data. Number of peptides identified for each protein in replicate CID and decision tree experiments are shown together with phosphopeptide numbers identified in phosphorylation experiments.

Table S6 Putative tyrosine phosphorylation sites. Putative tyrosine phosphorylation sites are indicated (for DT CID and ETD spectra and DDNL CID spectra) and the difference in SLoMo score between a phosphotyrosine site localisation and an alternative phosphoserine/threonine localisation is included. All PSMs matching to contaminating human proteins are coloured in red font. The total number of PSMs contributing evidence to each tyrosine phosphorylation site is indicated.

Supplementary data to this article can be found online at http://dx.doi.org/10.1016/j.jprot.2014.03.010.

## Conflicts of interest

All authors declare no financial/commercial conflicts of interest.

## Figures and Tables

**Table 1:** Summary of spectra acquired and results of Mascot and Mascot Percolator analysis.

		Replicate 1	Replicate 2	Replicate 3	Total	Assignment rate
*A) Decision tree*						
CID	Spectra acquired	39,904	38,227	39,290	117,421	
	MP PSMs (*q* 0.01)	17,595	17,796	17,870	53,261	45.4
	**MP PSMs (PEP 0.01)**	**15,305**	**15,327**	**15,230**	**45,862**	**39.1**
	Mascot (*q* 0.01)	10,803	11,996	11,617	34,416	29.3
**ETD**	Spectra acquired	15,804	15,356	15,239	46,399	
	MP PSMs (*q* 0.01)	6977	6758	6577	20,312	43.8
	**MP PSMs (PEP 0.01)**	**6156**	**6107**	**5860**	**18,123**	**39.1**
	Mascot (*q* 0.01)	5416	5173	5069	15,658	33.7

*B) Data dependent neutral loss triggered ETD*						
CID	Spectra acquired	36,865	37,446	36,006	110,317	
	MP PSMs (*q* 0.01)	17,191	16,627	15,870	49,688	45.0
	**MP PSMs (PEP 0.01)**	**14,918**	**14,291**	**13,974**	**43,183**	**39.1**
	Mascot (*q* 0.01)	11,374	10,561	10,269	32,204	29.2
ETD	Spectra acquired	16,286	14,338	18,353	48,977	
	MP PSMs (*q* 0.01)	7367	7954	8642	23,963	48.9
	**MP PSMs (PEP 0.01)**	**6081**	**6663**	**7396**	**20,140**	**41.1**
	Mascot (*q* 0.01)	5314	5617	5976	16,907	34.5

Results in bold were used to generate the final phosphoproteome datasets.

**Table 2:** Comparison of CID and ETD in DT and DDNL experiments.

		Replicate 1	Replicate 2	Replicate 3	Total
*A) Decision tree*					
CID	PSMs (PEP < 0.01)	15,305	15,327	15,230	45,862
	PhosphoPSMs (PEP < 0.01)	10,567	10,962	10,984	32,513
	% phosphoPSMs	69%	72%	72%	71%
	Unique phosphopeptide sequences	2525	2610	2674	3272
ETD	PSMs (PEP < 0.01)	6156	6107	5860	18,123
	PhosphoPSMs (PEP < 0.01)	4396	4229	4299	12,924
	% phosphoPSMs	71%	69%	73%	71%
	Unique phosphopeptide sequences	1115	1087	1076	1361
	CID/ETD overlap (unique phosphopeptides)	628	633	638	835
	Overlap %	20.86%	20.67%	20.51%	21.99%
	Total phosphoPSMs	14,963	15,191	15,283	45,437
	Total unique phosphopeptides	3010	3062	3110	3798

*B) DDNL*					
CID	PSMs (PEP < 0.01)	14,918	14,291	13,974	43,183
	PhosphoPSMs (PEP < 0.01)	8962	9667	9307	27,936
	% phosphoPSMs	60%	68%	67%	65%
	Unique phosphopeptide sequences	2342	2538	2443	3272
ETD	PSMs (PEP < 0.01)	6081	6663	7396	20,140
	PhosphoPSMs (PEP < 0.01)	5862	5429	6666	17,957
	% phosphoPSMs	96%	81%	90%	89%
	Unique phosphopeptide sequences	2061	1957	2200	2996
	CID/ETD overlap (unique phosphopeptides)	1646	1611	1744	2458
	Overlap %	59.70%	55.92%	60.20%	64.55%
	Total phosphoPSMs	14,824	15,096	15,973	45,893
	Total unique phosphopeptides	2757	2881	2897	3808

DT/DDNL overlap					3197
DT/DDNL total unique phosphopeptide sequences					4409
Overlap					72.51%

**Table 3:** (A) Phosphorylation site assignment for high confidence spectral pairs. (B): Summary of localised phosphorylation sites in DDNL and DT strategies.

A	
*DDNL CID/ETD spectral pairs with matching phosphopeptide sequences*	
All (no PEP threshold)	18,874
PEP < 0.01 in either CID or ETD	17,802
PEP < 0.01 in both CID and ETD	12,618

Phosphorylation site matching between CID/ETD spectral pairs (PEP < 0.01 in both CID and ETD)	12,618
Default Mascot phosphorylation site assignment	10,902
No SLoMo score cut off	9063
*SLoMo FLR < 0.05*	*9049*
Breakdown of SLoMo FLR < 0.05	
Site localised in both CID and ETD spectra	7334
Site localised in ETD spectrum only	1696
Site localised in CID spectrum only	19
Site match in CID and ETD (SLoMo FLR > 0.05)	14

*Phosphorylation sites that do not match between CID/ETD pairs (PEP < 0.01 in both CID and ETD)*	
*SLoMo FLR < 0.05*	*2573*
Site localised in ETD spectrum only	1900
Site localised in CID spectrum only	415
Site localised in CID and ETD spectra (different sites)	258
*Total spectral pairs with localised phosphorylation sites* *(SLoMo FLR < 0.05)*	*11,622*


B			
	Phospho-PSM's	Site localised	% site localised
		SLoMo FLR < 0.05	
*DDNL triggered ETD*			
CID/ETD spectral pairs^a^	12,618	11,622	92.10
CID spectra only	15,321	7650	49.93
ETD spectra only	5336	4104	76.91
Total^b^	45,893	34,998	76.26

*Decision tree*			
CID spectra	32,513	17,873	54.97
ETD spectra	12,924	8344	64.56
Total	45,437	26,217	57.70

Results in italics were used to generate the final phosphoproteome datasets with an FLR of <5%.

a Spectral pairs.

b Spectral pairs counted individually.
