# Supplementary material for: Confident and sensitive phosphoproteomics using combinations of collision induced dissociation and electron transfer dissociation
Source: J Proteomics. 2014 May 30;103(100):1–14. doi: 10.1016/j.jprot.2014.03.010 (PMC4047622; doi:10.1016/j.jprot.2014.03.010)

Figure S1: Extended comparison of phosphorylation site assignment methods. The performance of Mascot (default rank 1 assignment), Mascot delta score, Ascore, PhosphoRS and SLoMo (with a range of score thresholds) for phosphorylation site assignment was tested using a human phosphopeptide library analysed by both CID and ETD [22]. The specificity and sensitivity of each approach can be compared by looking at the proportion of incorrectly assigned spectra (green) and correctly assigned spectra (red) to the total spectra (dark blue) and the percentage of those scored (light blue). Score threshold reported to give a 5% and a 1% FLR are included. The measured false localisation rate of 5% is indicated by a dashed red line.

Figure S2. Schematic of acquisition strategies. In DT experiments, peptides are selected for CID or ETD fragmentation based on their charge and m/z in order to exploit performance characteristics of each fragmentation method to maximise peptide identifications. In data dependent neutral loss triggered ETD experiments, all peptides are fragmented using CID and if prominent neutral loss peaks (indicative of phosphopeptides) are observed then the same precursor ion is selected for fragmentation using ETD. In this way, phosphopeptides are targeted for ETD fragmentation when there is evidence in the CID scan the peptide in question is phosphorylated.

Figure S3. Overlap of phosphopeptide identifications by CID and ETD and between DT and DDNL experiments. Numbers of non-redundant phosphopeptides (base sequences) identified by CID and ETD in DT experiments are shown in A, by DDNL experiments in B and the overlap between identifications in DDNL and DT experiments in C. The overlap of CID and ETD phosphopeptide identifications is low (21.9%) in the DT method and high (64.5%) in the DDNL

method. Similar numbers of unique phosphopeptides were identified by DT and DDNL methods with an overlap of 72.5%.

Figure S4. Charge state and m/z distributions for CID and ETD spectral pairs in DDNL experiments. Heat maps highlighting the numbers of phosphopeptide identifications specific to ETD (A), CID (B) and the difference between ETD and CID (C) for CID/ETD spectral pairs in DDNL experiments. A PSM is considered specific to a fragmentation method if it was identified with a PEP of less than 0.01 in one method and above this threshold in the alternative fragmentation method. It can be seen that CID performs best for lower charge state and higher m/z, particularly over 1000 m/z and ETD for higher charge state and lower m/z, particularly for 3+ and 4+ peptides between 500-800 m/z.

Figure S5. Proteome profiling using CID-only and DT acquisition. The overlap in protein identifications for triplicate CID experiments and triplicate DT experiments are shown in A and B, respectively. The overlap in protein identifications between the CID only approach and the DT approach was 82% (C).

Figure S1: Extended comparison of phosphorylation site assignment methods

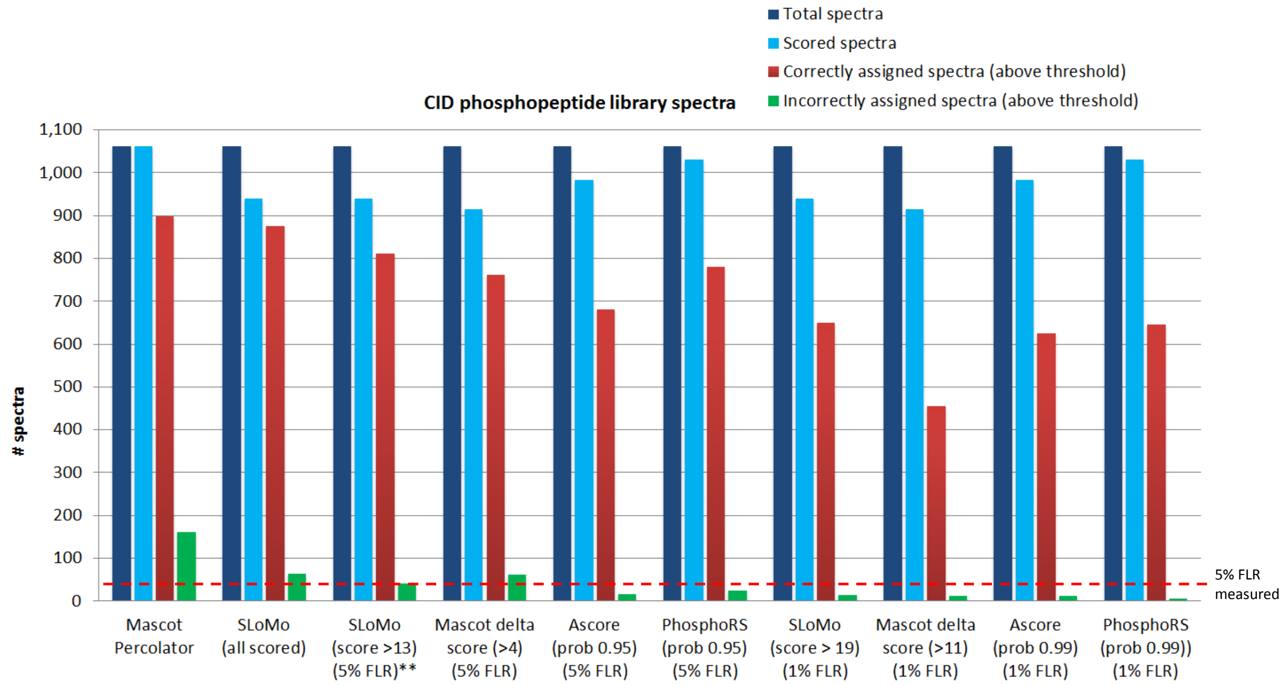

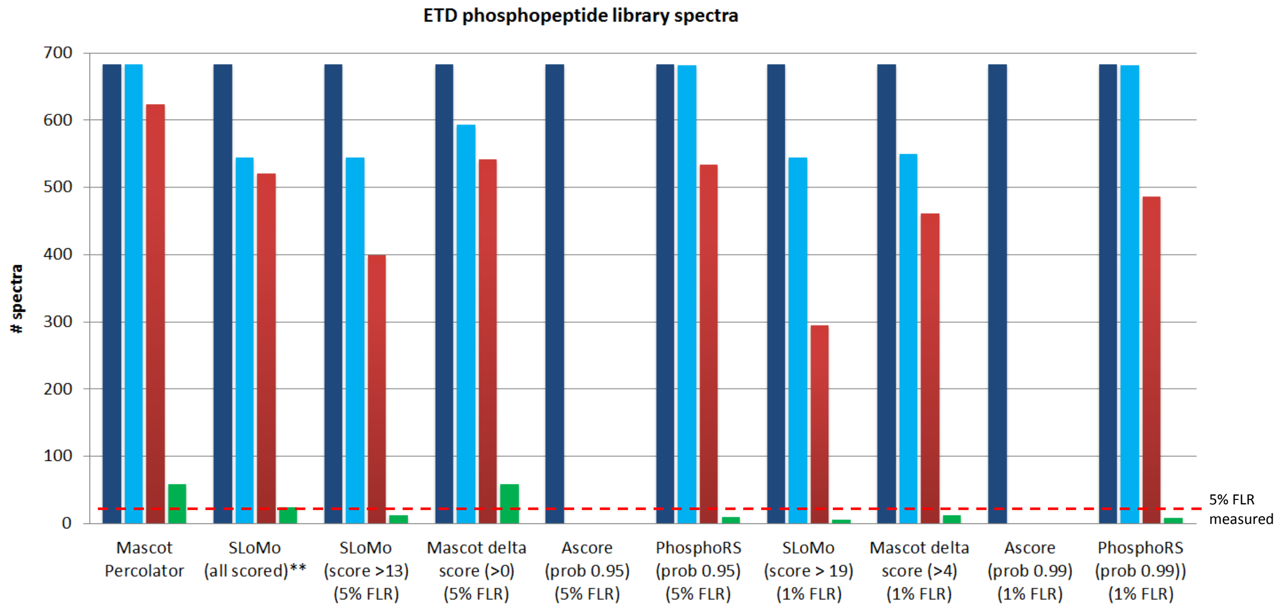

Figure S2. Schematic of acquisition strategies

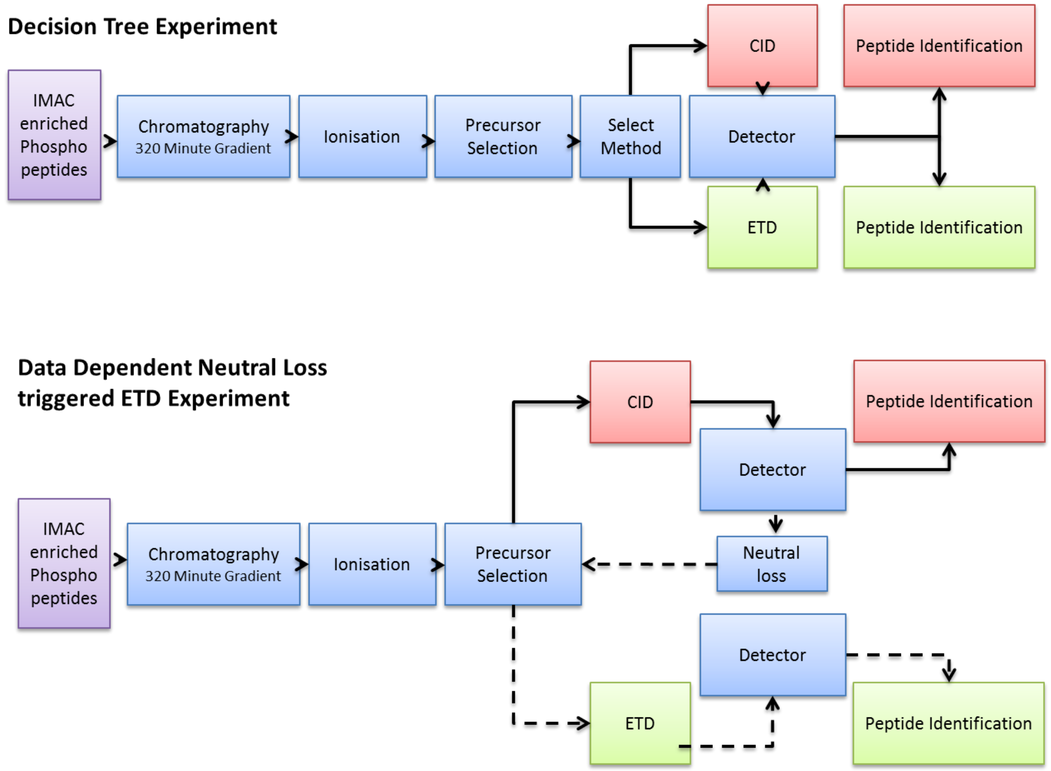

Figure S3. Overlap of phosphopeptide identifications by CID and ETD and between DT and DDNL experiments.

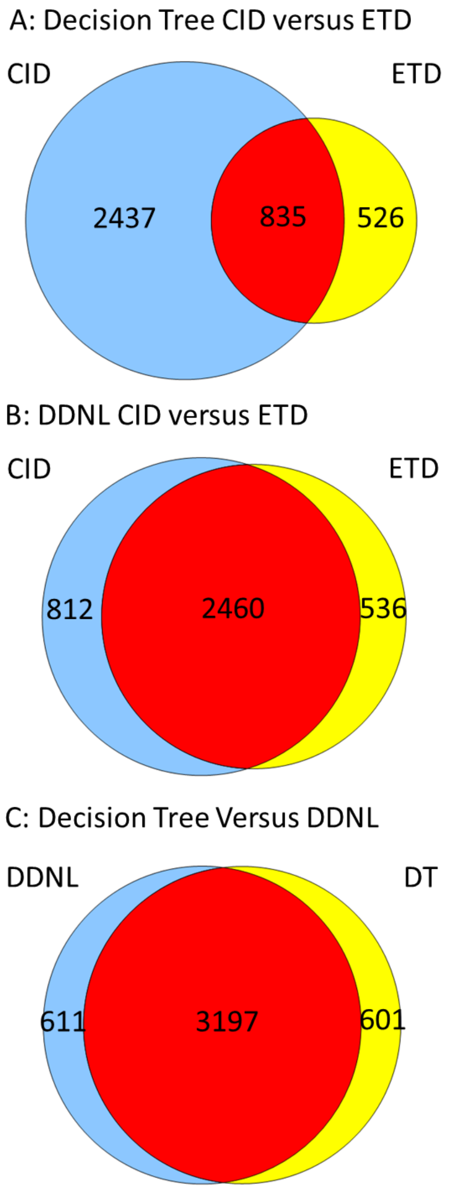

Figure S4. Charge state and m/z distributions for CID and ETD spectral pairs in DDNL experiments.

A: 0.01 PEP in ETD only

m/z	Precursor charge state 2	3	4	5	6	7
400	7	21	0	0	0	0
500	52	116	62	12	0	0
600	167	539	134	61	6	0
700	305	821	238	115	12	1
800	360	688	224	60	27	15
900	341	424	255	72	22	4
1000	3	268	73	20	1	0
1100	0	176	11	5	0	0
1200	0	39	0	0	2	0
1300	0	0	8	7	0	0
1400	0	2	2	0	0	0
1500	0	0	2	2	0	0

A: 0.01 PEP in CID only

m/z	Precursor charge state 2	3	4	5	6	7
400	44	29	0	0	0	0
500	194	4	0	0	0	0
600	459	16	0	0	0	0
700	299	112	21	7	1	0
800	255	395	39	7	0	2
900	216	730	63	27	0	1
1000	981	413	196	10	2	0
1100	478	552	81	1	2	0
1200	213	256	73	2	0	0
1300	74	62	25	0	0	0
1400	4	9	0	0	0	0
1500	3	0	0	0	0	0

C: ETD only minus CID only PSMs

m/z	Precursor charge state 2	3	4	5	6	7
400	-37	-8	0	0	0	0
500	-142	112	62	12	0	0
600	-292	523	134	61	6	0
700	6	709	217	108	11	1
800	105	293	185	53	27	13
900	125	-306	192	45	22	0
1000	-978	-145	-123	10	-1	0
1100	-478	-376	-70	4	-2	0
1200	-213	-217	-73	0	0	0
1300	-74	-62	0	0	0	0
1400	-4	-7	0	0	0	0
1500	-3	0	0	0	0	0

Figure S5. Proteome profiling using CID-only and decision tree acquisition

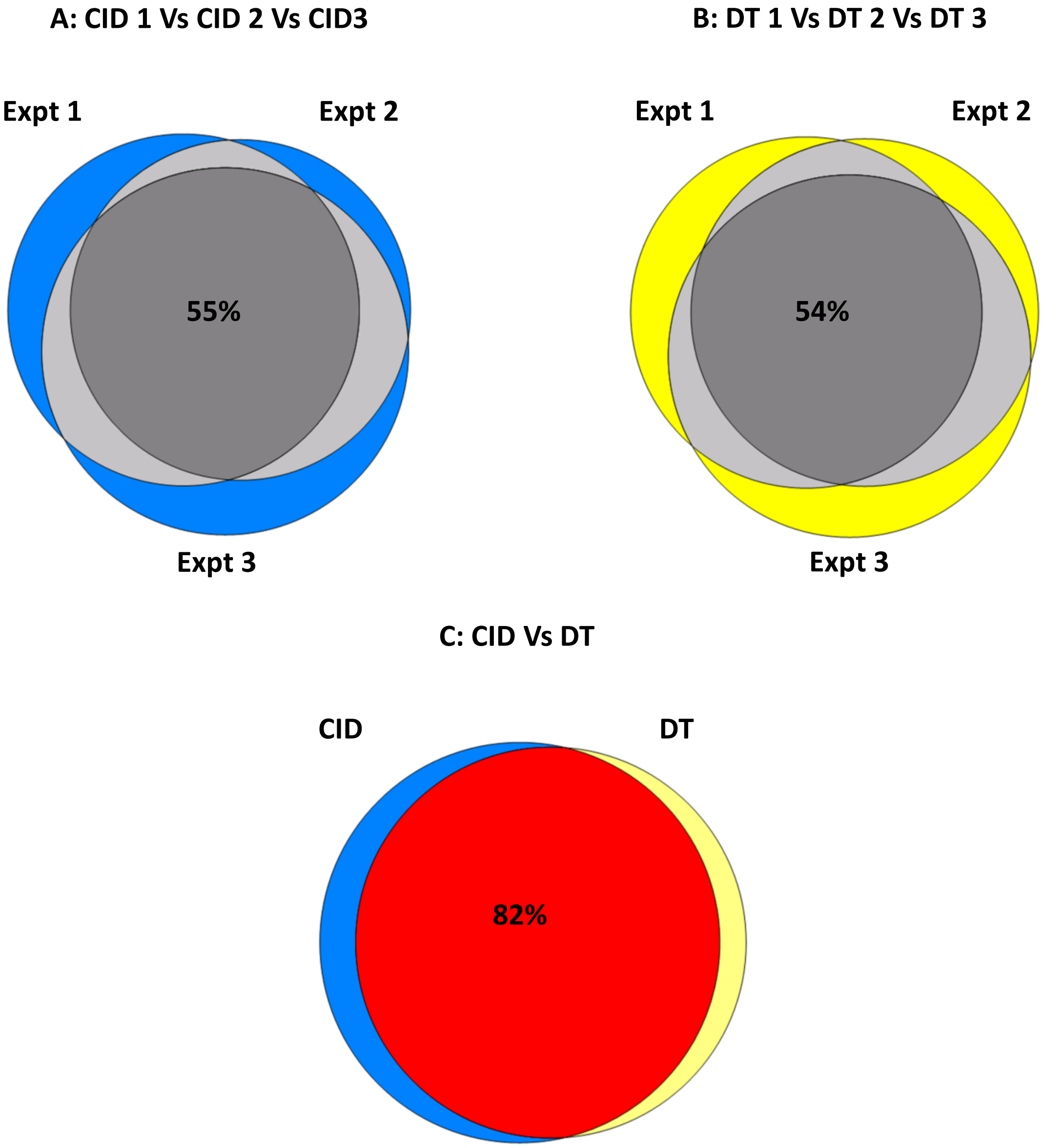

Supplement: Supplementary file 1 — Supplementary Figures. [file mmc1.pdf]
